# Preoperative management using inhalation therapy for pulmonary complications in lung cancer patients with chronic obstructive pulmonary disease

**DOI:** 10.1007/s11748-017-0761-5

**Published:** 2017-03-09

**Authors:** Kyoshiro Takegahara, Jitsuo Usuda, Tatsuya Inoue, Takayuki Ibi, Akira Sato

**Affiliations:** Department of Thoracic Surgery, Nippon Medical School, 1-1-5 Sendagi, Bunkyo-ku, Tokyo, 113-8603 Japan

**Keywords:** Chronic obstructive pulmonary disease, Long-acting beta-agonist, Long-acting muscarinic antagonist, Lung cancer, Complications

## Abstract

**Objective:**

This study aimed to investigate whether perioperative inhalations of long-acting beta-agonists (LABAs) or long-acting muscarinic antagonists (LAMAs) might decrease the incidence of postoperative complications in lung cancer patients with chronic obstructive pulmonary disease (COPD).

**Methods:**

We retrospectively analyzed 108 patients with COPD who underwent pulmonary resections for primary lung cancer at our hospital between January 2013 and January 2016 to determine the association between the incidence of postoperative complications (e.g., prolonged air leakage and pneumonia) and the use of LABAs or LAMAs.

**Results:**

Thirty patients with COPD experienced postoperative complications (27.8%): Fourteen patients had prolonged air leakages (more than 7 days), ten patients developed pneumonia. The frequency of these postoperative pulmonary complications was significantly higher among the patients with COPD (24/108 cases, 22.2%), compared with the frequency among non-COPD patients (15/224 cases, 6.7%). Inhaled bronchodilators, such as LAMA or LABA, were prescribed for 34 of the 108 patients with COPD; the remaining 74 patients were not treated with bronchodilators. Pulmonary complications were significant lower among the LAMA or LABA users (3/34 cases, 8.8%) than among the untreated COPD patients (21/74 cases, 28.4%).

**Conclusion:**

For lung cancer patients with COPD, preoperative management using LABA or LAMA bronchodilators and smoking cessation can reduce the frequency of postoperative pulmonary complications after surgical lung resection. LAMA or LABA inhalation might be useful for not only perioperative care, but also for the long-term survival of COPD patients after surgery.

## Introduction

Chronic obstructive pulmonary disease (COPD) causes impaired pulmonary function as a symptom of occlusive disorder and is associated with a significantly high incidence of postoperative pulmonary complications, such as pneumonia, acute bronchitis, and atelectasis. Many patients with COPD have nonspecific airway hyperreactivity, suggesting the possible presence of bronchospasm or latent respiratory tract infection. To reduce the risk of postoperative complications, efforts should be made to alleviate peripheral airway obstruction and to reduce airway secretion [1, 2]. In Japan, with its aging society, the number of patients with lung cancer complicated with COPD is expected to further increase, while an increased incidence of multiple lung cancers makes the perioperative management of surgically treated patients with lung cancer very important [3]. Because the prognosis of patients with lung cancer complicated with COPD is reportedly poor, it is important to provide respiratory care not just during the perioperative period after pneumonectomy, but also for longer periods [4, 5]. The combination of smoking cessation, respiratory rehabilitation, and the use of long-acting muscarinic antagonists (LAMAs) or long-acting beta-agonists (LABAs) has been reported to alleviate postoperative complications in patients with lung cancer complicated with COPD [4, 6–11]. The causal factors for airway obstruction in COPD are an irreversible decrease in elastic alveolar recoil, peripheral airway obstruction, and reversible contraction of the airway smooth muscle. The relaxation of airway smooth muscle is necessary for the resolution of airway obstruction. To treat the pathological symptoms of COPD, anticholinergics, which prevent acetylcholine from binding to muscarinic receptors, and beta-2 agonists are used. Thus, acetylcholine and the muscarinic receptor system play important roles in the pathogenesis of COPD [2, 12, 13].

Recently developed LABAs are superior to the previous ones in terms of their bronchodilation efficacy. The fourth edition of the Guidelines for the Diagnosis and Treatment of Chronic Obstructive Pulmonary Disease mentions that LAMAs and LABAs are comparable with respect to their usefulness for inhalation therapy and recommends inhalation therapy with LAMAs or LABAs [13, 14]. In this study, we aimed to investigate whether the incidence of postoperative complications during the perioperative period could be reduced in patients with lung cancer complicated by COPD by not only providing smoking cessation services before surgery, but also by initiating the inhalation of LABAs or LAMAs.

## Patients and methods

We defined ex-smokers as patients who had quit smoking at least 6 months before surgery; current smokers were defined as those who had quit smoking within 6 months before surgery. Patients with a smoking history and a forced expiratory volume in 1 s (FEV1.0) to forced vital capacity (FVC) ratio of less than 70% were classified as COPD patients; the other patients were classified as non-COPD patients. Of the 332 patients who underwent surgical resections for primary lung cancer at our hospital between January 2013 and January 2016, the medical records of 108 patients who had COPD and a smoking history were retrospectively analyzed to determine the association between the incidence of postoperative complications (e.g., prolonged pulmonary fistulae and pneumonia) and the use of LABAs or LAMAs. We recommended inhalations to all the patients based on the diagnostic criteria of COPD and used them for the patients who agreed to the recommendation. The medical records of the remaining 224 non-COPD patients were also examined for comparison purposes. In addition, the use of LABAs or LAMAs started from at least 1 month ago of the operation and continued after operation based on the fourth edition of the Guidelines for the Diagnosis and Treatment of Chronic Obstructive Pulmonary Disease.

## Results and discussion

Among the 108 COPD patients, there were 86 men and 22 women, with a mean age of 69.3 years (range 46–84 years). The mean Brinkman index was 1172.1 (range 50–3480). There were 45 current smokers and 63 ex-smokers. The mean FEV1.0/FVC was 61.4% (range 26.8–69.9%) (Table 1). The performed surgical procedures were a partial resection in 11 patients, a pulmonary segmentectomy in 3 patients, a lobectomy in 92 patients, and a pneumonectomy in two patients. The histological types were adenocarcinoma in 53 patients, squamous cell carcinoma in 38 patients, adenosquamous carcinoma in 5 patients, large cell neuroendocrine carcinoma in 3 patients, large cell carcinoma in 4 patients, small cell carcinoma in one patient, and pleomorphic carcinoma in 4 patients (Table 1). There were no surgical or in-hospital deaths. Postoperative complications were observed in 30 patients. The patients received wedge resection are not included in these 30 patients. Regarding the incidence of pulmonary complications, such as prolonged air leakage and pneumonia, 24 patients (22.2%) with COPD and 15 (6.7%) non-COPD patients experienced complications. The incidence was significantly higher among the COPD patients than among the non-COPD patients. No significance differences in the incidences of arrhythmia, wound infection, or other complications were observed between the COPD and non-COPD patients (Table 2). The incidence of prolonged air leakage and pneumonia was significantly lower among the 34 patients who received LABAs or LAMAs (3 patients, 8.8%) than among the 74 patients who did not receive these drugs (21 patients, 28.4%) (Table 3 ).

**Table 1:** Clinicopathological characteristics of the study population

Characteristics	
COPD(non-COPD)	108 (224)
Age (years)	69.3 (46–84)
Male/female	86/22
Smoking (BI)	1172.1 (50–3480)
Current/ex-smoker	45/63
FEV1.0/FVC	61.4 (26.8–69.9)
c-stage(IA/IB/IIA/IIB/IIIA)	49/24/16/7/12
Operation methods	
Wedge resection	11
Segmentectomy	3
Lobectomy	92
Pneumonectomy	2
Histological type	
Adeno	53
Squamous	38
Large	4
Small	1
LCNEC	3
Others	9

**Table 2:** Postoperative complications

	COPD(108)	Non-COPD(224)	*P* value
Prolonged air leakage	14 (13.0%)	9 (4.0%)	0.0055
Pneumonia	10 (9.3%)	6 (2.7%)	0.0188
IP	0	2	0.8196
Arrhythmia	2	4	1
Chylothorax	2	2	0.831
Wound infection	2	3	1

**Table 3:** (A) Postoperative complications of COPD patients. (B) Postoperative complications of current smoker

A	LABA/LAMA	No medications	*P* value
Prolonged air leakage + Pneumonia	3/34(8.8%)	21/74(28.4%)	0.0433

	Current smoker	Ex-smoker	*P* value
Prolonged air leakage + Pneumonia	9/45(20.0%)	15/63(23.8%)	0.8144

B	LABA/LAMA	No medications	*P* value
Prolonged air leakage + Pneumonia	3/24(12.5%)	6/21(28.6%)	0.3315

The COPD patients included 45 current smokers; 24 of these patients had undergone smoking cessation therapy for at least 1 month before surgery, respiratory rehabilitation, and the inhalation of LABAs or LAMAs. Three of the twenty-four patients (12.5%) who underwent inhalation therapy as part of their preoperative management developed prolonged air leakages or pneumonia, whereas six of the twenty-one current smokers (28.6%) who did not 66 receive inhalation therapy developed these pulmonary complications. The incidence of pulmonary complications was lower among current smokers who had started inhalation therapy as part of their preoperative management; however, there was no significant difference statistically (Table 3).

In the present study, examining lung cancer patients with COPD, preoperative management that included the inhalation of LABAs or LAMAs reduced the frequency of postoperative pulmonary complications after surgical resection. For the reason why complications decrease by preoperative inhalation therapy, we think that perioperative sealing tests go well and that drainage of the sputum from peripheral airway goes well by inhalation therapy. Other possibility was that the inhalation of LABA or LAMA prevented the pneumonia at the peripheral lung by anti-inflammatory effect. We think that further detailed examination will be necessary in future. Whereas, it was reported that inhalation therapy is not necessarily effective as Yamanashi et al. reported. This report shows that there was no significant difference about inhalation therapy using corticosteroids (ICSs) [15]. As for the recent LABA, effects come out early and effects are high [9, 13, 14]. However, we think that the accumulation of cases is necessary for comparison.

LABA and LAMA inhalants can be adapted for the management of perioperative care. For elderly patients with lung cancer, especially those with concurrent COPD, not only perioperative but also long-term respiratory care, including the administration of LABAs or LAMAs, might be necessary. Further prospective studies may be needed for the long-term survival of lung cancer patients with COPD after surgical resections.
